# Enhancing endometrial receptivity in FET cycles: exploring the influence of endometrial and subendometrial blood flow along with endometrial volume

**DOI:** 10.3389/fmed.2024.1260960

**Published:** 2024-04-08

**Authors:** Vajihe Hazari, Fatemeh Sarvi, Ashraf Alyasin, Marzieh Agha-Hosseini, Sedigheh Hosseinimousa

**Affiliations:** ^1^Department of Obstetrics and Gynecology, Rooyesh Infertility Center, Birjand University of Medical Science, Birjand, Iran; ^2^Department of Obstetrics and Gynecology, Tehran University of Medical Science, Tehran, Iran

**Keywords:** Doppler ultrasound, FET cycles, endometrial blood flow, IVF, endometrial receptivity

## Abstract

**Introduction:**

Fetal health and a receptive and healthy endometrium are two essential factors in achieving successful implantation. If the endometrium is unreceptive, postponing the transfer cycle to a suitable time can enhance the chances of pregnancy. This study aims to assess the impact of endometrial and sub-endometrial blood flows measured by Doppler ultrasound, as well as endometrial volume, on endometrial receptivity in frozen embryo transfer (FET) cycles.

**Methods:**

112 patients with a mean age of 33.93 ± 4.93 years underwent *in vitro* fertilization (IVF). Serum β-hCG level was used to confirm pregnancy, and among the participants, 50 (44.6%) achieved pregnancy after IVF.

**Results:**

The study results revealed a significant difference in endometrial blood flow between the pregnant and non-pregnant groups, with a higher pregnancy rate observed in participants exhibiting multi-focal and spare endometrial blood flows (*p* < 0.05). Furthermore, there was a notable association between endometrial blood flow and pregnancy outcome, as indicated by higher ongoing pregnancy rates in those with multi-focal and spare endometrial blood flows (*p* < 0.05). However, no significant differences were observed in endometrial variables such as volume, length, width, thickness, and pattern between the pregnant and non-pregnant groups. Additionally, contextual parameters showed no significant relationship with pregnancy outcome (*p* > 0.05). The study also found that endometrial measurement indices did not have a significant impact on pregnancy outcomes, with no significant differences observed between the groups (*p* > 0.05).

**Conclusion:**

In conclusion, endometrial blood flow is crucial for a successful pregnancy after IVF, while the predictive value of the endometrial volume is limited for pregnancy outcomes.

## Introduction

Embryo transfer (ET) is a critical stage in achieving a successful pregnancy in women undergoing *in vitro* fertilization (IVF). Implantation, a complex process, necessitates a coordinated and interactive relationship between the blastocyst and the endometrium. This intricate interaction can be compromised by suboptimal embryo quality or impaired endometrial receptivity, resulting in failed implantation (1–3). Research indicates that the embryo alone is attributed to approximately one-third of implantation failures (3), while the remaining two-thirds are associated with impaired endometrial receptivity or disrupted embryo-endometrium interaction (4).

Despite the utilization of various techniques such as preimplantation genetic testing (PGT), metabolomics, time-lapse photography, and optimization of embryo-endometrium interaction through receptivity methods, their impact on the success of *in vitro* fertilization (IVF) has been limited (5). Consequently, further studies are warranted to comprehensively evaluate additional factors beyond embryo quality and conventional endometrial markers that potentially influence the success of embryo transfer (ET). In addition to the requirement for a healthy and high-quality embryo, the presence of a healthy, functional, and receptive endometrium is crucial for achieving successful ET in humans. Determining the precise timing of endometrial receptivity, which represents the most critical period in human fertility and, consequently, the IVF cycle, is a fundamental consideration for achieving favorable outcomes in IVF. Numerous tests and trials have been employed to investigate this matter; however, some of these methods have proven to be costly, invasive, and associated with uncertain outcomes (5).

Endometrial ultrasound is a commonly used non-invasive procedure to assess endometrial receptivity during the IVF process. Adequate blood supply to the endometrium is considered essential for successful implantation, making the evaluation of endometrial blood flow a topic of significant interest in recent years. Doppler ultrasound of uterine arteries alone may not accurately reflect the actual blood flow to the endometrium. A more reliable and objective measurement of endometrial and sub-endometrial blood flows can be achieved through the use of three-dimensional power Doppler ultrasound (3D-PDU). However, conflicting results have been reported regarding the efficacy of these measurements in predicting pregnancy outcomes in the IVF process (6, 7).

Given the accessibility, non-invasiveness, and cost-effectiveness of functional ultrasound imaging techniques, along with the recognized significance of endometrial parameters such as endometrial thickness, endometrial pattern, endometrial volume, and endometrial blood flow, this study aimed to assess the impact of endometrial and subendometrial blood flows, endometrial volume, and endometrial thickness on endometrial receptivity in frozen embryo transfer (FET) cycles.

## Materials and methods

### Study population

This prospective cohort study was conducted on infertile women who were referred to the infertility ward of Shariati Hospital and Omid Infertility Clinic in Tehran, Iran, for frozen embryo transfer (FET) between May and December 2019. A convenience sampling method was employed to select a sample size of 112 women who met the study’s inclusion criteria. These criteria included willingness to participate, age between 20 and 42 years, body mass index (BMI) less than 35, baseline follicle-stimulating hormone (FSH) level below 12, absence of endometrial abnormalities (such as polyps) or myometrial abnormalities (fibroids) or congenital uterine anomalies or Asherman’s syndrome (traumatic hypomenorrhea-amenorrhea), lack of endometriosis, and availability of one or two good-quality embryos. Exclusion criteria encompassed cases where transvaginal ultrasound (TVUS) images could not be obtained, instances of difficult embryo transfer, history of recurrent implantation failure (RIF), and unwillingness to continue cooperation in the follow-up process.

### Ethical considerations

The research proposal for this study was initially approved by the Research Council of Tehran University of Medical Sciences. Subsequently, it underwent a thorough review by the ethics committee of Tehran University of Medical Sciences and obtained approval with the ethics code IR.TUMS.MEDICINE.REC.1399.253. Prior to commencing the study, the study design was thoroughly explained to all participants, including the objectives, the receipt of routine treatment during the study, the absence of any interventions, the confidentiality of their identities, and the performance of a single Doppler ultrasound examination, which would only take a few minutes and be provided free of charge. Finally, written informed consent was obtained from all participants involved in the research.

### Data collection

To collect the necessary data for this study, the demographic and clinical information of all patients was initially extracted from their medical records. On the second or third day of the menstrual cycle, following a routine baseline ultrasound and confirmation of endometrial and ovarian function suppression (If the endometrial thickness ≤ 5 mm and there were no dominant follicles in both ovaries), hormone replacement therapy (HRT) was initiated using estradiol valerate (ESTRADIOL VALERATE ABURAYHAN 2MG TAB). The initial dosage of estradiol valerate was 2 mg twice daily, with subsequent adjustments based on the observed endometrial thickness on ultrasound. This standardized protocol was followed for all patients to prepare the endometrium. Serial ultrasound examinations were performed, and the dosage of estradiol was adjusted to achieve an endometrial thickness greater than 7 mm, as per the routine protocol. Endometrium with a thickness greater than 7 mm and displaying a multilayer pattern was selected for progesterone administration and designated for embryo transfer. On this day, patients were prescribed 800-mg vaginal progesterone suppositories (cyclogest 400 mg, Actoverco pharmaceutical factory). After six days of progesterone therapy, One or two good blastocyst was transferred.

On the day of embryo transfer, patients underwent ultrasound examination to assess and evaluate endometrial parameters such as thickness, pattern, volume, and blood flow. This comprehensive examination was conducted by an experienced physician prior to the embryo transfer procedure. Following the embryo transfer, patients continued to receive estradiol and progesterone (800-mg suppository and 50-mg ampoule, FERTIGEST ABURAYHAN 50MG AMP) We use them until 12 weeks. On the 14th day after embryo transfer, a pregnancy test was conducted, and the subsequent pregnancy outcomes were monitored for a duration of 12 weeks. Subsequently, the relationship between ultrasound parameters assessed on the day of embryo transfer and both the pregnancy rate and pregnancy outcomes was investigated.

### Ultrasound study

**Figure 1:**
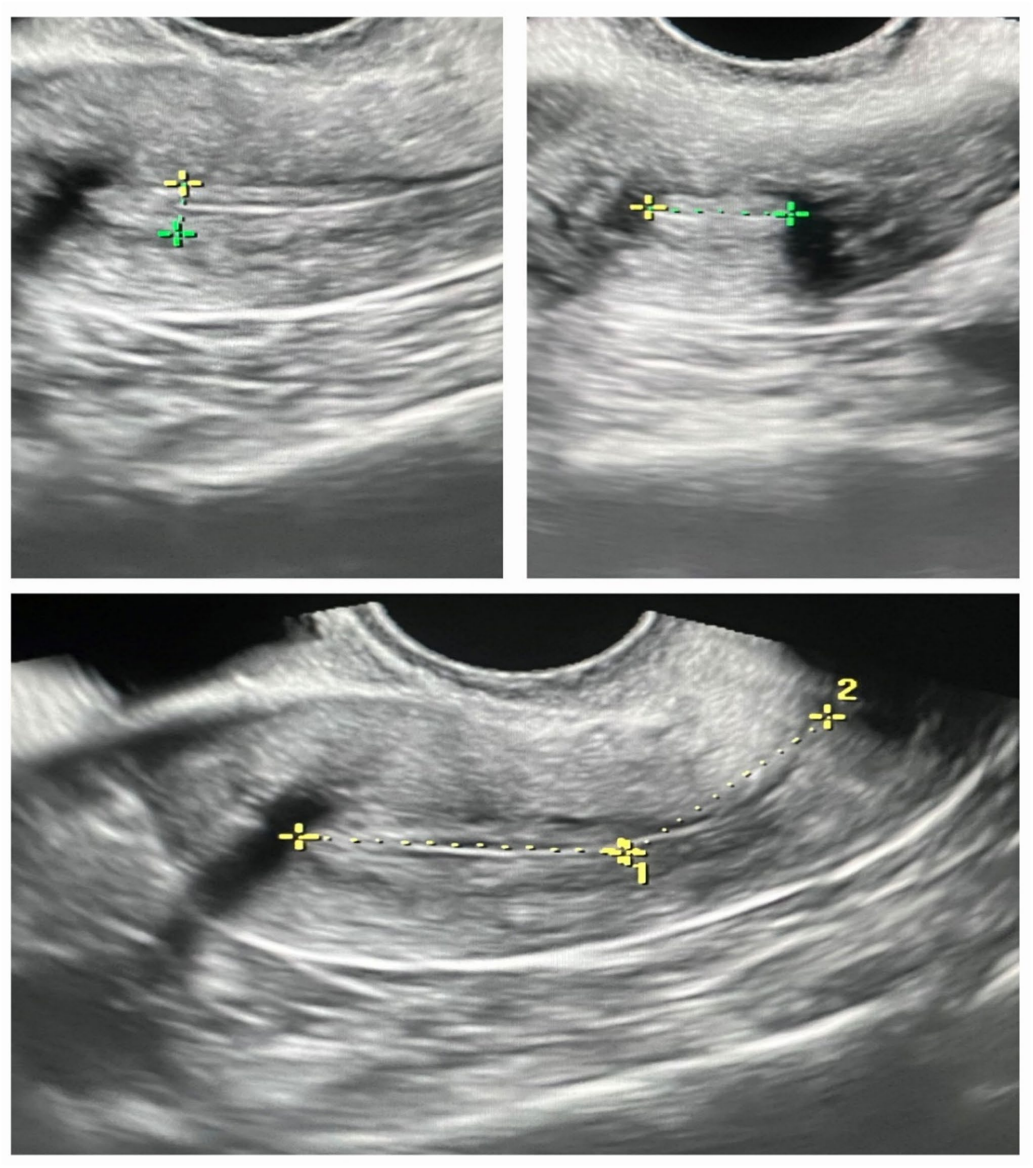
Comparison of endometrial measurements in pregnant and non-pregnant groups: length, width, thickness, and volume.

Thirty minutes prior to the embryo transfer, all patients underwent a comprehensive ultrasound examination (by MEDISONE D20) while in the lithotomy position and with an empty bladder vaginally. The uterus was visualized in the longitudinal section, allowing for the observation of the entire endometrial and sub-endometrial areas, ranging from the fundus to the internal orifice of the uterus. The subendometrial region, situated 10 mm beneath the myometrial-endometrial junction, was specifically assessed. We provided thickness, length, width as inputs for VOCAL software and the output of the software was the volume (Figure 1). Based on the observed blood flow, three groups were established: the absence of blood flow (absent), scattered blood flow (spare), and multifocal blood flow (multifocal). Morphologically, the endometrial pattern was further categorized into three groups: pattern A, characterized by a triple-line or multilayer endometrium; pattern B, exhibiting an isoechoic endometrium; and pattern C, presenting a hyperechoic endometrium.

### Embryo transfer

All embryo transfers were conducted using a Cook catheter by a skilled practitioner. The transfers were categorized into three groups based on their level of difficulty: (1) easy transfer, (2) successful transfer with the use of a mandrel, and (3) challenging and potentially traumatic transfer (8). Notably, none of the patients encountered a difficult transfer, as all individuals fell into either the easy transfer or successful transfer with a mandrel group (groups 1 and 2).

### Pregnancy outcomes

In this study, pregnancy outcomes were classified into the following categories: (1) Chemical pregnancy, indicated by a positive pregnancy test; (2) Clinical pregnancy, characterized by the presence of a gestational sac with a heartbeat observed on an ultrasound two weeks after the positive pregnancy test; (3) Ongoing pregnancy, denoting the continuation of pregnancy beyond 12 weeks; and (4) Ectopic pregnancy, identified by the presence of a gestational sac outside the uterus.

### Statistical analysis

The normal distribution of the data was evaluated using the Kolmogorov–Smirnov test. To compare means between two independent groups, the independent t-test was utilized for data with a normal distribution, while the Mann–Whitney test was employed for data with a non-normal distribution. For mean comparison among multiple independent groups, the one-way analysis of variance (ANOVA) was conducted for data with a normal distribution, whereas the Kruskal-Wallis test was employed for data with a non-normal distribution. Qualitative data was analyzed using the Chi-square test. All data were analyzed using SPSS version 21 software, with a significance level set at a *p*-value<0.05. Additionally, graphs were generated using Graphpad Prism version 3 software.

## Results

In this study, a total of 112 patients with a mean age of 33.93 ± 4.93 years underwent the IVF technique, and pregnancy confirmation was based on the serum β-hCG level. Among the 112 participating patients, 50 (44.6%) achieved pregnancy following IVF. Various variables were assessed in these patients after IVF, including age, BMI, duration of infertility, cause of infertility, AMH and FSH levels, number of ETs, and number of transferred embryos (Table 1). It was observed that none of these contextual variables showed a significant difference between the pregnant and non-pregnant groups of women (*p* > 0.05).

**Table 1:** Demographic and baseline variables in patients participating in the study.

Variables	βHCG			*p*-value
	Negative	Positive	Total	
	Mean (Stdv)	Mean (Stdv)	Mean (Stdv)	
	Frequency (%)	Frequency (%)	Frequency (%)	
Age (year)	34.04 (4.83)	33.80 (5.10)	33.93 (4.93)	0.793
BMI (kg/m2)	26.04 (4.19)	26.42 (3.60)	26.21 (3.92)	0.604
Infertility duration (year)	4.59 (2.63)	5.33 (2.80)	4.92 (2.72)	0.158
AMH (ng/ml)	4.96 (5.31)	5.90 (5.41)	5.38 (5.35)	0.360
FSH (mIU/mL)	4.50 (2.05)	5.03 (2.75)	4.74 (2.39)	0.254
**Cause of infertility**				
Male factor	25 (56.8)	19 (43.2)	44 (100)	0.757
Ovarian	7 (50)	7 (50)	14 (100)	
Tubular	7 (77.8)	2 (22.2)	9 (100)	
U/E	12 (48)	13 (52)	25 (100)	
Male factor + ovarian	9 (56.3)	7 (43.7)	16 (100)	
Male factor + tubular	2 (50)	2 (50)	4 (100)	
**Number of transfers**				
First transfer	28 (57.1)	21 (42.9)	49 (100)	0.443
Second transfer	34 (54)	29 (46)	63 (100)	
**Embryos (Blastocyst) transferred**				
One	19 (63.3)	11 (36.7)	30 (100)	0.209
Two	43 (52.4)	39 (47.6)	82 (100)	

**Figure 2:**
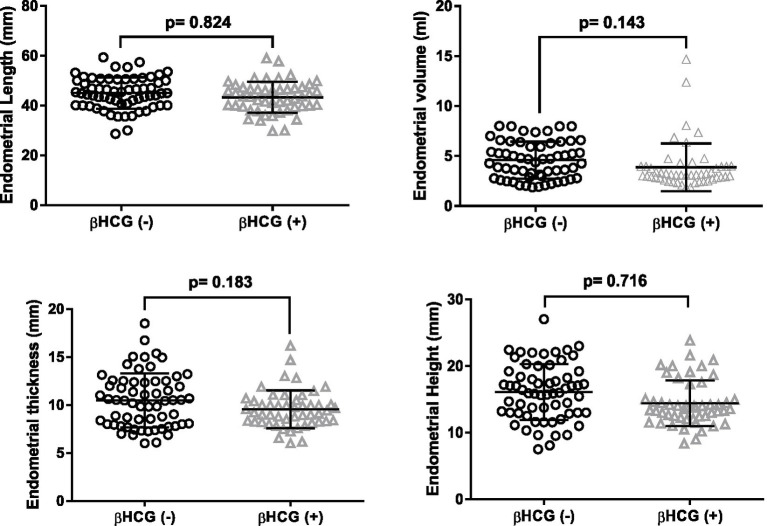
Endometrial measurements in patients stratified by pregnancy outcomes: length, width, diameter, and thickness.

In this study, we assessed and compared the endometrial parameters, which included endometrial volume, length, width, and thickness, in two groups of women undergoing IVF: pregnant and non-pregnant (Figure 2). The results indicated that there were no significant differences observed in endometrial volume, length, width, and thickness between the two groups of women (*p* > 0.05).

Mean endometrial *volume* was 4.4 mL in pregnant and was 3.8 mL in non pregnant. There was no significant different (*p* > 0.05). In our study, the mean endometrial *thickness* in pregnant cases was 9.5 mm and that of non-pregnant cases 11 mm with *p* value (0. 183).

Furthermore, we examined the endometrial blood flow and pattern among the women included in this study (Table 2). The endometrial blood flow was classified into three categories: absent, spare, and multi-focal, and the patients were grouped accordingly.

**Table 2:** Analysis of endometrial blood flow and endometrial pattern in study participants.

Variables	βHCG			*p*-value
	Negative	Positive	Total	
	Frequency (%)	Frequency (%)	Frequency (%)	
**Endometrial blood flow**				
Absent	28 (90.3)	3 (9.7)	31 (100)	<0.001
Spare	23 (62.2)	14 (37.8)	37 (100)	
Multi focal	11 (25)	33 (75)	44 (100)	
**Endometrial patterns**				
Multi-layer	17 (63)	10 (37)	27 (100)	0.284
Isoechoic	23 (46.9)	26 (53.1)	49 (100)	
Hyperechoic	22 (61.1)	14 (38.9)	36 (100)	

Based on the findings presented in Table 2, the endometrial pattern did not exhibit a significant difference between pregnant and non-pregnant women (*p* > 0.05). However, the endometrial blood flow demonstrated a significant disparity between the two groups of pregnant and non-pregnant women. Notably, a higher pregnancy rate was observed among participants with multi-focal and spare endometrial blood flows (*p* < 0.05).

**Figure 3:**
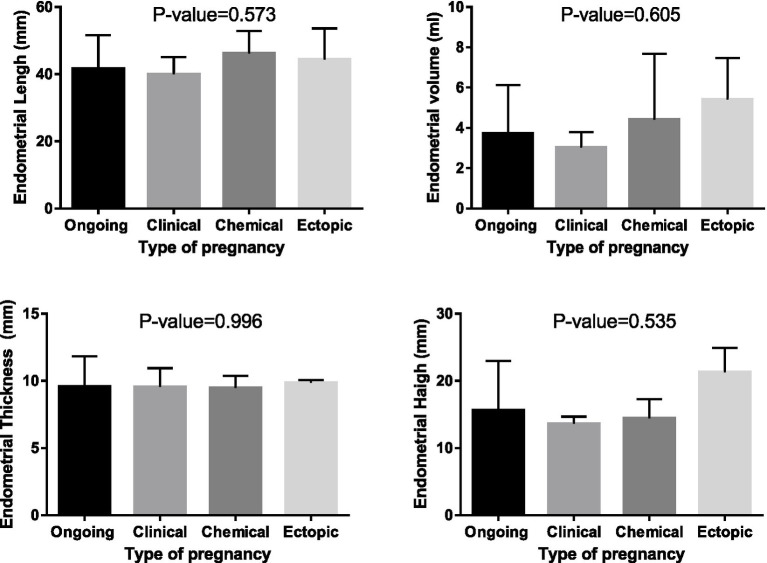
The technique how to measure endometrial volume, length, width, and thickness.

After confirming pregnancy, pregnant women underwent two-dimensional Doppler ultrasound to analyze the ultrasound findings. The study then examined the role of underlying variables and endometrial factors in determining the type of pregnancy (refer to Tables 3, 4 and Figure 3). The results revealed a significant relationship between endometrial blood flow and pregnancy outcome, indicating higher ongoing pregnancy rates in individuals with multi-focal and spare endometrial blood flows (*p* < 0.05). However, other underlying variables did not show a significant association with pregnancy outcome (*p* > 0.05). Additionally, Figure 3 demonstrated that endometrial parameters had no impact on pregnancy outcomes, with no significant differences observed between the groups (*p* > 0.05).

**Table 3:** Association between study variables and pregnancy outcome.

Variables	Sonography results					*p*-value
	Ongoing pregnancy	Clinical pregnancy	Chemical pregnancy	Ectopic pregnancy	Total	
	Mean (Stdv)	Mean (Stdv)	Mean (Stdv)	Mean (Stdv)	Mean (Stdv)	
Age (year)	33.34 (4.9)	34.4 (6.1)	35 (6.02)	35 (4.94)	33.93 (4.93)	0.808
BMI (kg/m2)	26.01 (3.04)	25.97 (5.02)	27.99 (5.11)	28.6 (0.14)	26.21 (3.92)	0.439
Infertility duration (year)	5.3 (2.88)	5.4 (1.51)	4.62 (3.11)	8.5 (0.7)	4.92 (2.72)	0.390
AMH (ng/ml)	5.54 (5.01)	8.4 (6.01)	6.41 (7.2)	3.9 (4.3)	5.38 (5.35)	0.681
FSH (mIU/mL)	4.88 (2.77)	5.82 (3.01)	4.79 (2.71)	6.5 (3.53)	4.74 (2.39)	0.779

**Table 4:** Association between study variables and pregnancy outcome.

Variables	Sonography results					*p*-value
	Ongoing pregnancy	Clinical pregnancy	Chemical pregnancy	Ectopic pregnancy	Total	
	Frequency (%)	Frequency (%)	Frequency (%)	Frequency (%)	Frequency (%)	
**Cause of infertility**						
Male factor	14 (73.7)	2 (10.5)	3 (15.8)	0 (0)	19 (100)	0.215
Ovarian	4 (57.1)	1 (14.3)	1 (14.3)	1 (14.3)	7 (100)	
Tubular	2 (100)	0 (0)	0 (0)	0 (0)	2 (100)	
U/E	10 (76.9)	2 (15.4)	0 (0)	1 (7.7)	13 (100)	
Male Factor + ovarian	5 (74.1)	0 (0)	2 (28.6)	0 (0)	7 (100)	
Male Factor + tubular	0 (0)	0 (0)	2 (100)	0 (0)	2 (100)	
**Number of transfers**						
First transfer	16 (76.2)	1 (4.8)	4 (19)	0 (0)	21 (100)	0.415
Second transfer	19 (65.5)	4 (13.8)	4 (13.8)	2 (6.9)	29 (100)	
**Embryos (Blastocyst) transferred**						
One	7 (63.6)	3 (27.3)	1 (9.1)	0 (0)	11 (100)	0.153
Two	28 (71.8)	2 (5.1)	7 (17.9)	2 (5.1)	39 (100)	
**Endometrial blood flow**						
Absent	2 (66.7)	1 (33.3)	0 (0)	0 (0)	3 (100)	0.019
Spare	5 (35.7)	2 (14.3)	6 (42.9)	1 (7.1)	14 (100)	
Multi Focal	24 (84.8)	2 (6.1)	2 (6.1)	1 (3)	29 (100)	
**Endometrial patterns**						
Multi-layer	8 (80)	0 (0)	2 (20)	0 (0)	10 (100)	0.312
Isoechoic	19 (73.1)	3 (11.5)	4 (15.4)	0 (0)	26 (100)	
Hyperechoic	8 (57.1)	5 (10)	2 (14.3)	2 (14.3)	17 (100)	

## Discussion

The objective of this study was to assess the impact of endometrial and subendometrial blood flows, as well as endometrial volume and thickness, on endometrial receptivity in FET-HRT cycles. The results of the study revealed a significant disparity in endometrial blood flow between pregnant and non-pregnant women, with a higher pregnancy rate observed in women exhibiting multi-focal and spare blood flows. Moreover, a notable association was found between endometrial blood flow and pregnancy outcome, indicating a higher ongoing pregnancy rate in women with multi-focal and spare blood flows. However, the endometrial variables of volume, length, width, thickness, and pattern did not exhibit a significant difference between the pregnant and non-pregnant groups. Furthermore, contextual variables displayed no significant relationship with pregnancy outcome. Additionally, the endometrial parameters had no impact on pregnancy outcomes, with no significant difference observed between the groups.

This study aimed to assess endometrial blood flow in patients undergoing IVF. The results revealed that the majority of patients (39.3%) exhibited multi-focal blood supply, followed by spare and absent blood supply. Based on these findings, a significant correlation was observed between endometrial blood flow and the pregnancy rate. This finding aligns with the study conducted by Chien et al., which examined the impact of endometrial blood flow on pregnancy outcomes following IVF and reported that it could serve as a predictor of successful pregnancy (9). Thus, the results of the present study are consistent with prior research.

Ng et al. (10) presented results that further support a significant association between pregnancy outcomes after IVF and endometrial blood flow. Similarly, in a meta-analysis conducted by Wang et al. (11), the findings indicated a positive and significant impact of endometrial blood flow on pregnancy rates following IVF.

Furthermore, this study revealed a significant correlation between endometrial blood flow and ultrasound findings. Specifically, the results demonstrated that the majority of individuals with ongoing pregnancies (84.8%) exhibited multi-focal blood supply in the endometrium. This finding aligns with the outcomes of a prospective study conducted by Souidan and Salama (12), which highlighted the ability of endometrial and sub-endometrial blood flows to predict ultrasound findings or, in other words, pregnancy outcomes with a sensitivity of 57.5 and specificity of 61.6. Similar to our study, Souidan and Salama also reported the highest rate of ongoing pregnancy to be among women with multi-focal blood flow in the endometrium. In a study conducted by Sardana et al. (7), it was observed that groups with higher endometrial blood supply exhibited improved pregnancy outcomes. These findings are consistent with the results reported by Martins et al. (13), Ragheb et al. (14), and Ziadi et al. (15). However, Mayer et al. (8) presented contrasting findings, as they reported no significant differences in FI, VI, VFI, endometrial, and submandibular blood flows between the pregnant and non-pregnant groups. Contrary to the findings of Kim et al. (16) and Sudha Prasad et al. (17), which suggested no significant association between endometrial blood flow and pregnancy, this study’s results highlight a different perspective. The findings of this study showed that patients with improved endometrial blood flow exhibited higher rates of pregnancy and successful embryo transfer. Consequently, there exists a strong correlation between endometrial blood flow and the likelihood of successful embryo transfer and development, underscoring the diagnostic significance of endometrial blood flow in predicting outcomes of IVF-ET. In this study, endometrial volume was measured using a 2D probe, resulting in a recorded volume of 4.4 mL in pregnant women and 3.8 mL in non-pregnant women. However, the findings of this study indicated that endometrial volume did not exhibit a significant association with pregnancy or pregnancy outcomes. Likewise, no significant relationships were observed between endometrial length, width, and height in women and their pregnancy or pregnancy outcomes. It is important to note that previous studies have reported diverse findings regarding the predictive value of endometrial volume for IVF outcomes. However, several studies, including those conducted by Mayer et al. (8), Aboulghar et al. (18), and others (19–21), have demonstrated that endometrial volume serves as a predictive factor for pregnancy in patients undergoing embryo transfer. Furthermore, some studies have indicated that the predictive value of endometrial volume may be lower when comparing 2D and Doppler ultrasound parameters (22, 23). In a meta-analysis examining postpartum women, it was found that endometrial volume did not differ significantly between pregnant and non-pregnant individuals (24). Similarly, a meta-analysis conducted in 2017 revealed that endometrial volume did not exhibit a significant difference between pregnant and non-pregnant women (25). Similar findings were reported by Souidan and Salama (12), supporting the results of the present study. In a meta-analysis conducted by Boza et al. (26), it was determined that endometrial volume had no significant impact on pregnancy outcomes. The conflicting results observed in previous research can be attributed to variations in the methods employed to measure endometrial volume, as well as potential methodological limitations. Studies conducted between 1999 and 2005 utilized the trapezoid formula or volume acquisition or rotational methods to measure endometrial volume, while the Virtual Organ Computer-aided Analysis (VOCAL) imaging program with less or optimal plane measurements was introduced after 2005 (26). The results of this study align with those of Boza et al., with the distinction that endometrial volume was measured using a similar two-dimensional method in this study, whereas a three-dimensional method was utilized in Boza’s study. Therefore, it can be suggested that variations in study outcomes across different research investigations may be influenced by human and environmental factors. or It seems that the endometrial volume may related to individual changes between the race and size of the patients.

In our study, the mean endometrial thickness was measured to be 9.5 mm in pregnant women and 12.3 mm in non-pregnant women. These findings are consistent with the studies conducted by Ragheb et al. (14), Mayer et al. (8), Ziadi et al. (15), Souidan and Salama (12), and Kim et al. (16), which also reported no significant difference in endometrial thickness and similar pregnancy rates. However, these results contrast with the findings of studies by Martins et al. (13) and Shivtare et al. (27). Of course, all our patients had an endometrial thickness of more than 7 cm on the day of starting progesterone.

Regarding the endometrial pattern, our study observed a higher frequency of isoechoic and hyperechoic patterns compared to the multilayer pattern. However, no significant difference was observed in the pregnancy rate between the multilayer pattern and the higher isoechoic and hyperechoic patterns.

Finally, the endometrial blood flow plays a significant role in enhancing the occurrence and sustainability of pregnancy following IVF rather than other endometrial variables such as volume, length, width, thickness, and pattern, to explain the possible reason for the differences could be that, Endometrial blood flow may affect the reactions between endometrium and embryo and Endometrial quality may be more important than its volume and dimensions.

Based on the findings of this study, it was observed that among the underlying variables considered, only AMH exhibited an inverse relationship with endometrial blood flow, while no significant associations were found between other factors and endometrial or subendometrial blood flows. Furthermore, this study investigated the impact of women’s age on endometrial and subendometrial blood flows measured by Doppler ultrasound during IVF treatment. The results revealed that women’s age did not have a significant effect on any of the Doppler flow indices in both the endometrial and subendometrial regions. These findings align with the results reported by Ragheb et al. (14) and Ng et al. (10), providing consistent evidence regarding the lack of influence of women’s age on Doppler flow indices in the endometrial and subendometrial areas. In this study, 2D ultrasound was utilized to measure endometrial parameters. The results of this study indicate that 2D ultrasound findings, which accurately predict endometrial blood supply and its association with pregnancy outcomes, demonstrate its potential effectiveness in facilitating successful IVF procedures. With the advancements in ultrasound technology, the application of color Doppler imaging (CDI) has become prevalent in evaluating endometrial blood flow. CDI offers several advantages, including heightened sensitivity to slow blood flow, reduced dependence on angles, and the provision of clearer images with less blurring. Furthermore, this method offers practicality while providing precise information about the endometrial condition in a non-invasive manner (28). In this study, the measurement of endometrial volume further corroborated that the results obtained from 3D ultrasound were entirely consistent with our findings. Consequently, in situations where state-of-the-art technology may not be readily available, 2D ultrasound can serve as a suitable tool for assessing uterine and endometrial parameters.

This study demonstrates several strengths, including the rigorous implementation of inclusion and exclusion criteria. Moreover, the recruitment of embryos with top-quality blastocysts further enhances the strength of the research design. Additionally, to minimize inter-observer variability, all ultrasounds conducted on the day of embryo transfer were performed by the same physician. Furthermore, the expertise of an experienced physician in performing all embryo transfers ensures consistency and mitigates potential technical differences that could impact the study outcomes. This study had a few limitations, including a small sample size and the utilization of a 2D ultrasound probe that was available at the study center. Although the 2D ultrasound results closely approximated the findings of 3D ultrasound, employing more up-to-date devices may yield even more precise and quantitative measurements of endometrial parameters. Another limitation of the study pertained to the cost associated with ultrasound, which was overcome through the center’s participation, thereby mitigating this potential constraint.

## Conclusion

Based on the findings of this study, it can be inferred that endometrial blood flow plays a significant role in enhancing the occurrence and sustainability of pregnancy following IVF. As a result, it is advisable to incorporate the assessment of endometrial blood flow as a routine examination before embryo transfer. It is recommended that future studies with larger sample sizes employ 3D Doppler ultrasound to further investigate this area. Additionally, it would be valuable to design studies exploring interventions that promote increased uterine blood flow, such as aspirin, prednisolone, vaginal sildenafil, L-arginine, lifestyle modifications, and other factors known to enhance implantation. These investigations would contribute to advancing our understanding and developing effective strategies to improve pregnancy outcomes.

## Data availability statement

The raw data supporting the conclusions of this article will be made available by the authors, without undue reservation.

## Ethics statement

The studies involving humans were approved by the research proposal for this study was initially approved by the Research Council of Tehran University of Medical Sciences. Subsequently, it underwent a thorough review by the ethics committee of Tehran University of Medical Sciences and obtained approval with the ethics code IR.TUMS.MEDICINE.REC.1399.253. The studies were conducted in accordance with the local legislation and institutional requirements. Written informed consent for participation in this study was provided by the participants’ legal guardians/next of kin.

## Author contributions

VH: Conceptualization, Writing – original draft, Writing – review & editing. FS: Conceptualization, Writing – original draft. AA: Writing – original draft. MA-H: Formal analysis, Writing – review & editing. SH: Data curation, Writing – review & editing.
